# Systematic interactome mapping of acute lymphoblastic leukemia cancer gene products reveals EXT-1 tumor suppressor as a Notch1 and FBWX7 common interactor

**DOI:** 10.1186/s12885-016-2374-2

**Published:** 2016-05-26

**Authors:** Sarah Daakour, Leon Juvenal Hajingabo, Despoina Kerselidou, Aurelie Devresse, Richard Kettmann, Nicolas Simonis, Franck Dequiedt, Jean-Claude Twizere

**Affiliations:** Laboratory of Protein Signaling and Interactions, Molecular Biology in Diseases Unit, GIGA-Research, University of Liège, Liège, B-4000 Belgium; Laboratoire de Bioinformatique des Génomes et des Réseaux (BiGRe), Université Libre de Bruxelles (ULB), Bruxelles, B-1050 Belgium

**Keywords:** Acute lymphoblastic leukemia, Cancer genes, Interactome, Notch pathway, EXT1

## Abstract

**Background:**

Perturbed genotypes in cancer can now be identified by whole genome sequencing of large number of diverse tumor samples, and observed gene mutations can be used for prognosis and classification of cancer subtypes. Although mutations in a few causative genes are directly linked to key signaling pathways perturbation, a global understanding of how known cancer genes drive oncogenesis in human is difficult to assess.

**Methods:**

We collected available information about mutated genes in Acute Lymphoblastic Leukemia (ALL). Validated human protein interactions (PPI) were collected from IntAct, HPRD and BioGRID interactomics databases, or obtained using yeast two-hybrid screening assay.

**Results:**

We have mapped interconnections between 116 cancer census gene products associated with ALL. Combining protein-protein interactions data and cancer-specific gene mutations information, we observed that 63 ALL-gene products are interconnected and identified 37 human proteins interacting with at least 2 ALL-gene products. We highlighted exclusive and coexistence genetic alterations in key signaling pathways including the PI3K/AKT and the NOTCH pathways. We then used different cell lines and reporter assay systems to validate the involvement of EXT1 in the Notch pathway.

**Conclusion:**

We propose that novel ALL-gene candidates can be identified based on their functional association with well-known cancer genes. We identified *EXT1*, a gene not previously linked to ALL via mutations, as a common interactor of NOTCH1 and FBXW7 regulating the NOTCH pathway in an FBXW7-dependend manner.

**Electronic supplementary material:**

The online version of this article (doi:10.1186/s12885-016-2374-2) contains supplementary material, which is available to authorized users.

## Background

The identification of genes responsible for oncogenesis is a major goal in cancer research. These genes are mostly defined as “altered genes directly promoting malignant progression”. After three decades of molecular cancer research, different strategies have been used to define the cancer genetic landscape. The catalogue of somatic mutations in cancer (COSMIC) (http://cancer.sanger.ac.uk) is a comprehensive resource of somatic mutations in human cancer samples curated from published studies and cancer genomes sequencing [1]. As of May 31, 2015, COSMIC reports a set of 572 genes, called the cancer gene census, for which mutations are associated with cancer development. In cancer samples, it is challenging to analyze and prioritize sequential mutation accumulation events, which occur in oncogenes and tumor suppressors genes. The mutations that provide a selective growth advantage in any step of tumorigenesis (initiation, clonal expansion, tumor formation) are known as driver mutations. Out of 572 cancer gene census, about 140 genes including 71 tumor suppressors and 54 oncogenes are well-accepted as cancer driver genes because mutations in those genes promote tumorigenesis [2]. These numbers are not static and should increase as more cancer genomes are sequenced. Other approaches are being used to identify novel candidate cancer genes including Genome Wide Association Studies (GWAS) for the identification of cancer-associated loci [3], in vivo transposon mutagenesis screens in mice “sleeping beauty technology”, for genes potentially implicated in tumorigenesis [4–6], and protein-protein interactions screens of gene products targeted by oncogenic viruses [7–9]. Integrating information from all the above resources in a “guilt-by-association” model that also considers interacting partners of cancer-associated gene products allowed prioritization of ~ 3000 genes potentially associated with cancer [10]. However, analyzing variations of mutations in time and space in different cancer types and subtypes (e.g., what driver genes are important for what cancer type at what stage) has been challenging. Few studies led to the discovery of a number of genes implicated in specific tumor types. As an example, children medulloblastoma tumor samples exhibit an average of 11 gene alterations compared to 55–121 in adult tumors [11], whilst lung and colorectal cancers require only 3 driver gene mutations [12]. In liquid tumors such as leukemia and lymphomas, it believed that, one of the most prevalent category of mutations involving cancer driver genes are chromosomal rearrangements such as BCR-ABL1 in chronic mylogenous leukemia (CML) [13], fusions involving nucleopins 98 and 124 and MLL gene fusions in acute myelogenous leukemia (AML) [2, 14, 15], and TEL-AML1 and TCF3-PBX1 in acute lymphoblastic leukemia (ALL) [16–18]. These gene fusions alone are often insufficient and may require additional genetic perturbations for leukemogenesis [19, 20].

We previously showed that protein-protein interactions (PPI) data could be used for interpretation of expression profiles in cancer samples in order to identify and prioritize target genes and pathways [20]. Here, we used PPI mapping strategies to explore information on cancer genes frequently mutated in ALL. We highlighted mutated hub proteins interconnected in an ALL-cancer gene products network and identified novel interacting partners that link key ALL-cancer driver gene products [21, 22].

## Methods

### Databases and literature PPI curation

Information about genes containing mutations in their coding regions was retrieved from the COSMIC database, evaluated; organized and selected genes were submitted for experimental analysis. To establish a catalog of genes and mutations associated with Acute Lymphoblastic Leukemia (ALL), we used the version 71 of COSMIC, previously downloaded to a local server and we extracted data only related to ALL. We developed and implemented a procedure that automatically collects information and check the consistency of changes with the coding sequences and find the corresponding positions on clones from the human ORFeome (http://horfdb.dfci.harvard.edu/).

The retrieved information include details provided at either nucleotide or protein level (mutation syntax), sample id (portion of a tumor being examined for mutations), tissue from which the sample originated, histological classification of the sample and the Pubmed id of the article that published the study. For each gene tested for PPIs using the yeast two-hybrid, we identified mutations positions on the ORFeome clone by sequence alignment (BLAST) then we verified if the protein sequence has undergone modifications as described by mutation syntax.

Human PPIs were collected and verified from the different interactomics databases IntAct [23], HPRD [24] and BioGRID [25]. Only physical PPIs validated at least in two independent references or by two methods were considered as confident and maintained for the analysis.

### Network data analyses and visualization

Network analyses and visualization of protein-protein interactions were carried out with Cytoscape software, which is a free software for visualizing, modeling and analyzing molecular and genetic interaction networks. Due to its features, Cytoscape and its plugins provide a powerful tool kit allowing to answer specific biological questions using large amounts of cellular network and molecular profiling information [26]. In our maps, the nodes represent proteins that are connected with edges representing pairwise interactions extracted from interaction databases and from our Y2H experimental assay.

### Cell culture and transfection

HEK293, HeLa and HeLa Notch1∆E-eGFP cells were cultured in DMEM supplemented with 10 % fetal bovine serum (FBS), 2 mM glutamine and penicillin/streptomycin. The same medium was used for U2OS Tet-on flp-in cells bearing isogenic transgenes encoding Notch1-Gal4. As for K562-control and K562 expressing Dll4 cells, they were grown and maintained in RPMI 1640 supplemented with 15 % FBS and antibiotics. T-ALL cell lines were grown and maintained in RMPI containing 10–20 % FBS and supplemented with antibiotics.

HEK293 cells were DNA-transfected with polyethylenimine (PEI) purchased from Sigma, reagent was dissolved in water at 1 mg/ml and preserved at −80 °C. Transfection with PEI was performed on HEK cells cultured in DMEM at 80 % confluence. Medium was changed before transfection and cells were collected 24 h post-transfection.

HeLa cells and HeLaN1ΔE-eGFP cells were DNA-transfected with lipofectamine 2000 reagent (Invitrogen) according to manufacturer’s instructions and collected 24 h post-transfection.

SiRNA transfection was performed with Calcium Phosphate using ProFection Mammalian Transfection kit from Promega according to manufacturer’s instructions on cells cultured in DMEM at 40–50 % confluence. Medium was changed 24 h later and cells were collected 48 h post-transfection.

For experiments involving both DNA and siRNA transfections, siRNA-transfection was performed according to manufacturer’s instructions and 24 h later after changing medium cells were transfected with DNA using lipofectamin 2000 reagent (Invitrogen) and cells were collected 24 h post DNA-transfection.

For proteasomal degradation inhibition, cells were treated with 10 μg/ml MG132 for 6 h before being collected.

siRNA sequences:

- siEXT1: 5′-GGAUUCCAGCGUGCACAUUtt-3′
- siFBXW7: 5′- GCAUAGAUUUUAUGGUAAtt-3′
- siCtrl: 5′- GGCUGCUUCUAUGAUUAUGtt-3′

### qRT-PCR

Total RNA was extracted using GeneJET RNA Purification Kit (Thermo scientific), DNaseI-treated on the column (Thermo Scientific) and reverse-transcribed with random primers (Thermo scientific). qPCR was performed using SYBER Green detection from Roche and run on Lightcycler 480 (Roche). mRNA quantification was performed relative to GAPDH housekeeping gene. Relative expression levels were calculated for each gene using the ΔΔCt method.

### Plasmids

Open reading frames (ORF) encoding Notch1 partners (tested for Protein complementation assay) were obtained from human ORFeome v5.1 (center of cancer systems biology: CCSB) as entry clones. Human NICD plasmid, was obtained from Addgene. FBXW7α expressing vector was kindly provided by Dr. E. Dejardin from the laboratory of molecular immunology and signal transduction—(GIGA-ULg). ORFs that were not available from the hORFeome V5.1 (BRAF, HRAS, ABL1, JAK2 and SMARCB1 and NOTCH1 genes), were purshassed from Genecopea and cloned by Gateway recombination technology (Invitrogen) using specific primers flanked with the following AttB1 and AttB2 Gateway sites: 5′- GGGGACAACTTTGTACAAAAAAGTTGGCATG-3′ (AttB1) and 5′- GGGGACAACTTTGTACAAGAAAGTTGA-3′ (AttB2). These constructions were verified by PCR and sequencing.

Inserts from pDONR223 were transferred by LR cloning (Invitrogen) into different destination vectors: pAD-destCYH and pDB-dest the Y2H expression vectors, and pDEST1899 (flag tag), pDEST491 (YFP-tag) and pDEST-mcherry for mammalian expression studies.

### High-throughput yeast Two-hybrid (HT-Y2H)

We used the hORFeome version 5.1, a collection of human ORFs cloned from the Mammalian Gene Collection (MGC) resource, representing a resource of ORFs that can be transferred easily to any Gateway compatible destination vectors. This collection contains 15 483 ORFs representing almost half of the human genome. They are cloned into the pAD-dest-CYH and pDB-dest encoding the yeast Gal4 Activating and DNA-binding domains, respectively. The resulting individual clones were transferred into MATa Y8800 (pAD) and MATα Y8930 (pDB) *S. cerevisiae* strains. Twenty-one selected ALL-genes were screened for interactions with the hORFeome V5.1 as described in [27].

One pool of 21 AD of selected genes into Y8800 yeast strain was mated to each of the 15483 dB-ORFs Y8930 of the hORFeome v5.1 and each of ALL-genes-DB into Y8930 yeast strain was mated to 165 pools of 94 AD-ORFs of the hORFeome v5. One Y2H screening was performed in the reciprocal orientation, as described in [27]. Positive colonies for the GAL1:: HIS3 and GAL1:: ADE2 selective markers but negative for autoactivation were selected for PCR-amplification (Zymolyase 20 T from Seikagaku Biobusiness, and Platinum® Taq DNA Polymerase from Invitrogen) and identification of interacting proteins by sequencing of the respective AD- and DB-ORFs.

### Luciferase reporter assays

Cells were seeded in 24-well plates and transfected with 300 ng of either TP1 luciferase reporter plasmid (TP1-luc) or CBF1 reporter plasmid (CBF1-luc) and 30 ng of renilla Luciferase (R-Luc). Twenty-four hours post-transfection luciferase activity was measured in cell lysates.

U2OS N1-Gal4 cells they were transfected 300 ng of Gal4-firefly luciferase and 30 ng R-Luc reporter plasmid. After 24 h, K562 cells expressing Notch ligands DLL4 or K562 control cells were added to the transfected cells in the presence of tetracycline (2 μg/mL). After 24-h of coculture, luciferase activity was measured in cell lysates.

Cell lysis and luciferase assays were performed in triplicate using Dual-luciferase reporter assay system from Promega. Luciferase measurements were performed in 96 well plates using DLR automated machine. Firefly luciferase values were normalized to R-luc values and calculated ratio represent luciferase activity.

### Immunofluorescence and confocal microscopy

HeLa Notch1∆E-eGFP cells were seeded onto coverslips in 24-well plates and transfected with 1 μg of EXT1-mcherry plasmid using lipofectamine2000 (Invotrogen). Twenty-four hours post-transfection, cells were washed in warm PBS, fixed in 3,7 % PBS-paraformaldehyde for 20 min at room temperature, washed 3 times with PBS, and mounted on glass coverslips using ProLong Gold Antifade montant with DAPI (life technologies).

Slides were examined by confocal microscopy using the Nikon A1R confocal system and images processed with the IMAGI software.

### Protein complementation assay (PCA)

NICD and FBXW7 were cloned in pN1Gluc vector and pN2Gluc vectors respectively (for *Gaussia* luciferase 1 and 2) using the Gateway cloning technology. HEK293 cells were seeded in 24 well-plates at a concentration 5.10 ^4^ cell/well, then transfected with GL1 or/and GL2 plasmids and 24 h post-transfection, luciferase activity was measured on lysates transferred into 96-well plate using and automated machine DLR with *Renilla* luciferase substrate. Normalized luciferase ratio was calculated as follows: NLR = luciferase value GL1 + GL2/(luciferase value GL1 + luciferase value GL2). An interaction is considered positive or validated when NLR ≥ 3.5. Cell lysis and luciferase assays were performed in triplicate for each condition.

### EXT1 silencing in zebrafish

Transgenic zebrafish line Tp1bglob:eGFP line [28] were maintained according to EU regulations on laboratory animals. Knockdown experiments were performed by injecting embryos at the one- to two-cell stage with 10 ng of single splice-blocking morpholino designed specifically for both *EXT1* a and b orthologs.

### RNA sequencing

Total RNA was extracted from HeLaN1ΔE-eGFP cells (siCTRL, siEXT1, siFBXW7), quantified and tested for RNA quality controlled using Agilent 2100 bioanalyzer using the Eukaryote Total RNA Nano assay. Total RNA strands were used to generate libraries and sequenced by HiSeq2000 sequencer.

### Statistical analysis

Graph values are presented as mean +/− standard deviation, calculated on at least three independent experiments. Unless stated otherwise, significance was determined using a two-tailed Student’s *t*-test (comparison of means). *P*-value thresholds are depicted as follows; *: *p* < 0.05; **: *p* < 0.01; ***: *p* < 0.001 and ****: *p* < 0.0001.

To prioritize ALL-genes, we combined the ranking from separate results (rank per number of mutation, rank per number of samples, rank per degree) by using order statistics. First, ranks are divided by the total number of ranked genes and we calculated the Q statistic [29], which represents the probability of obtaining the observed ranks r by chance, calculated using joint cumulative distribution of order as:

$$ Q\left({r}_1,{r}_2\kern0.5em ,\dots, \kern0.5em {r}_N\right)\kern0.5em =\kern0.5em N!{V}_N\kern3em {V}_0\kern0.5em =\kern0.5em 1,{V}_k=\kern0.5em {{\displaystyle {\sum}_{i=1}^k\left(-1\right)}}^{i\kern0.5em -\kern0.5em 1}\frac{V_{k\kern0.5em -\kern0.5em i}}{i!}{r}_{N-k+1}^i $$

Where ri is the rank ratio for result i, N is the number of genes used.

## Results and Discussion

### Mutations associated to ALL in cancer gene census

**Fig. 1:**
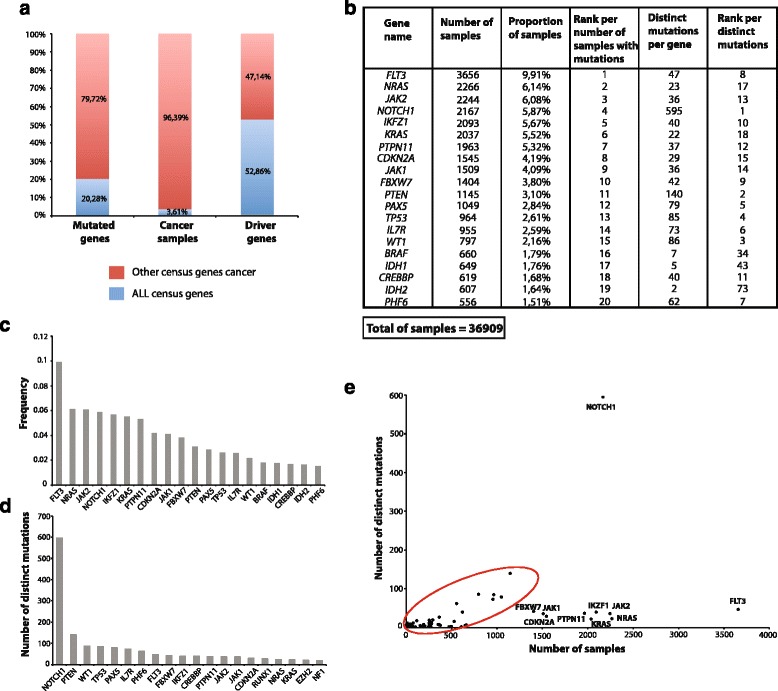
**a** Distribution of ALL census genes and other census genes according to number of mutations and number of samples and their distribution among driver genes. Red and bleu bars represent ALL census genes and other cancer census genes respectively (**b**) Mutations associated to ALL in cancer gene census. Frequency of mutations in the top 20 genes (36,909 ALL samples). The number and proportion of ALL samples in which gene mutations were detected are represented. **c** The 20 most frequently mutated genes in ALL samples; X-axis represents the proportion of samples where mutations were reported. **d** Number of distinct mutations per genes in ALL samples; X-axis represents the number of distinct mutations found in the coding sequences and Y-axis the top 20 genes with higher number of distinct mutations. **e** Occurrence of mutations per gene in ALL samples. Data source: COSMIC database

In order to identify cancer genes associated with acute lymphoblastic leukemia (ALL), we searched the COSMIC database version 71 and collected all available information about mutated genes in ALL samples. COSMIC V71 contains over 1,058,292 tumor samples containing over 2,710,449 coding mutations in 28,977 genes [1]. We found more than 2500 mutations in coding sequences of 366 genes that were reported in 36,909 ALL samples. In the COSMIC database, a set of 572 genes whose mutations are causally linked to oncogenesis, are called human Cancer Gene Census [30]. This set includes 140 genes well accepted as “cancer driver genes” because mutations in those genes directly promote tumorigenesis [2]. In ALL samples, we found that 20 % of the cancer gene census is affected by mutations in coding regions of 116 genes (Additional file 1: Table S1A). This high number of mutated genes is not due to over representation of ALL samples in COSMIC, as ALL samples count for about 3 % of tumor samples compiled in the COSMIC V71 (Fig. 1a). The “ALL-genes” set contains 74 well-known driver genes including 35 oncogenes and 39 tumor suppressor genes (TSG) (Additional file 1: Table S1B).

For each ALL-gene we extracted the number of samples as well as the number of distinct mutations. Figure 1b represents the top 20 frequently mutated genes among the 116 ALL-genes. Each gene that has at least 2 distinct mutations observed in at least 556 different samples, from a total of 36,909 ALL samples was examined. Seven genes were found mutated in more than 5 % of ALL samples, including genes encoding for FLT3 (9.9 %), NRAS (6.14 %), JAK2 (6.08 %), NOTCH1 (5.87 %), IKZF1 (5.67 %), KRAS (5.52 %) and PTPN11 (5.32 %) (Fig. 1b and c). We also ranked ALL-genes according to the number of distinct mutations found in ALL samples (Fig. 1b and d). The top ranked gene was NOTCH1 with 595 distinct mutations mostly found in its heterodimerization (HD) domain (63 % of mutations) and in its proline, glutamic acid, serine, threonine-rich (PEST) domain (27 % of mutations) (Additional file 2: Figure S1). Mutations in the HD domain that enhance NOTCH1 cleavage and nuclear translocation of the intracellular NOTCH1 protein (ICN), and mutations in the PEST domain that result in the stabilization of ICN, are gain-of-function mutations affecting the transcriptional activation of Notch1-target genes. The majority of these activating mutations were found in human T lymphocytes ALL (T-ALL) samples, as previously reported [31]. Other highly mutated ALL-genes include PTEN (140 distinct mutations) WT1 (86 distinct mutations), TP53 (85 mutations), PAX5 (79 mutations), and IL7R (73 mutations) (Fig. 1d).

**Fig. 2:**
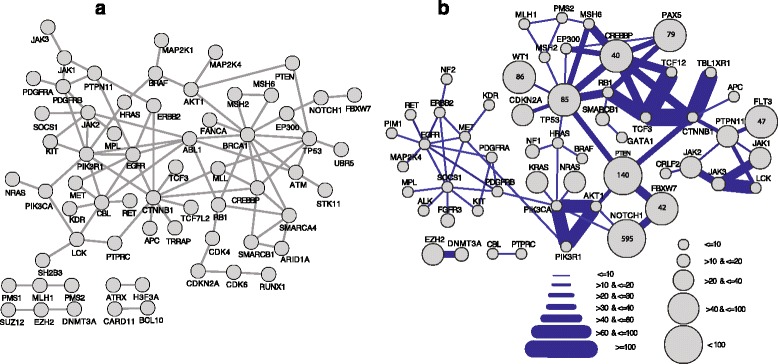
**a** Interactions between proteins mutated in ALL samples. Protein-protein interactions were extracted from three databases: IntAct, HPRD and BioGRID and only interactions reported with at least two publications, and detected by two experimental methods are represented in this map. **b** Co-occurrences of mutations in ALL samples. Nodes represent proteins associated with ALL, with an area proportional to the number of distinct mutations. Edges join pairs of interacting proteins for which mutations co-occur in the same samples. Edge widths are proportional to the number of samples with co-occurring mutations

For the majority of ALL-genes, the number of distinct mutations per gene correlated with the number of mutated samples (Fig. 1e, red circled), suggesting that a number of somatic mutations occurred randomly during oncogenesis, as previously observed for other types of cancers such as ovarian carcinoma or acute myeloid leukemia [32]. Another set of eight genes (*FBXW7*, *CDKN2A*, *PTPN11*, *IKZF1*, *JAK1, JAK2*, *KRAS* and *NRAS*) exhibit an average of 33 mutations in 1500–2500 examined ALL samples (Fig. 1e). These genes are characterized by similar mutations occurring in distinct ALL samples, suggesting their potential roles in clonal expansion of ALL. Two genes are outliers, and display many more mutations (*NOTCH1*) or are mutated in many more samples than average (*FLT3*). These larger numbers reflect the high rate of *NOTCH1* mutations specifically in T-ALL samples (99,9 % of *NOTCH1* mutations); and the involvement of FLT3 in childhood ALL, as previously described [33]. Interestingly, *NOTCH1* and *FLT3* mutations, mostly localized in two functional domains (HD and PEST for NOTCH1, juxtamembrane (JM) and tyrosine kinase (TKD) for FLT3) (Additional file 2: Figure S1), are found respectively in 1897 and 723 different patients (Fig. 1b), without co-occurrence in examined ALL samples (Fig. 2b).

### Interconnections between ALL-gene products

To analyze the connectivity between ALL-gene products, we collected protein-protein interactions (PPI) data from three databases: BioGRID [25], HPRD [24] and IntAct [23] and filtered all reported interactions between the 116 ALL-gene products. Figure 2a shows that 63 out 116 ALL-gene products are interconnected. We then prioritized ALL-gene products based on their degree of interconnectivity (Additional file 1: Table S2B). One of the top interconnected ALL-proteins is beta-catenin (encoded by CTNNB1 gene), which is a central hub in the Wnt/β-catenin signalling pathway and plays a crucial role normal haematopoiesis [34]. It has been shown that 50–85 % of the childhood T-ALL patients overexpress β-catenin [35], further supporting our finding that β-catenin is an important hub in ALL. Other Wnt/β-catenin signaling pathway members such as APC, TCF3 and TCF7L2 interacting with β-catenin, are part of our ALL-gene product set and were previously found differentially expressed in T-ALL patients [35].

Another example is PIK3R1 with 8 partners including PIK3CA that interacts with additional 4 ALL-gene products. PI3K members are essential effectors in the PI3K/AKT/mTOR signaling pathway, which is activated in a number of ALL samples [36]. Another example is ABL1 that interacts with 7 partners. The BCR-ABL1 fusion is the driver chromosomal rearrangement in chronic myeloid leukemia (CML) [37] and is also found in more than 20 % of ALL patients [38]. Mutations in *ABL1* gene were associated to different types of cancer [39].

### Co-occurrence of mutations in ALL-genes

We then explored the relationship between interacting genes based on the occurrence of mutations in the same ALL samples. We showed that, in addition to biophysical interactions, several ALL-gene products are mutated in the same patient samples, suggesting several ways of deregulating cancer pathways (Fig. 2b). As shown on Fig. 2b, our analysis revealed that ALL samples could be classified into 4 distinct groups of affected pathways, based on co-occurrence of mutations in important cancer driver genes: PI3K/AKT and NOTCH pathways, JAK and RAS pathways, Wnt/β-catenin and the cell cycle, and the transcriptional regulation pathways. Interestingly, protein phosphatases PTEN and PTPN11, and proteins important in genome maintenance and chromatin modification P53 and CREB binding protein are centrals and connect with deregulated pathways through different set of mutations (Additional file 1: Table S3).

Combining all the above criteria: frequency of mutations in individual cancer genes in ALL samples, number of distinct mutations and their pattern in ALL-gene (Fig. 1 and Additional file 2: Figure S1), interconnections between ALL-gene products and co-occurrence of mutated genes in the same samples (Fig. 2), we prioritized ALL-genes and suggest that *TP53*, *NOTCH1*, *CREBBP*, *PTEN*, *EGFR*, *JAK2*, *ABL1*, *PTPN11, CBL* and *EP300* are the top 10 ALL driver genes (Additional file 1: Table S4).

### Functional associations between ALL-gene products and their partners in the human proteome

**Fig. 3:**
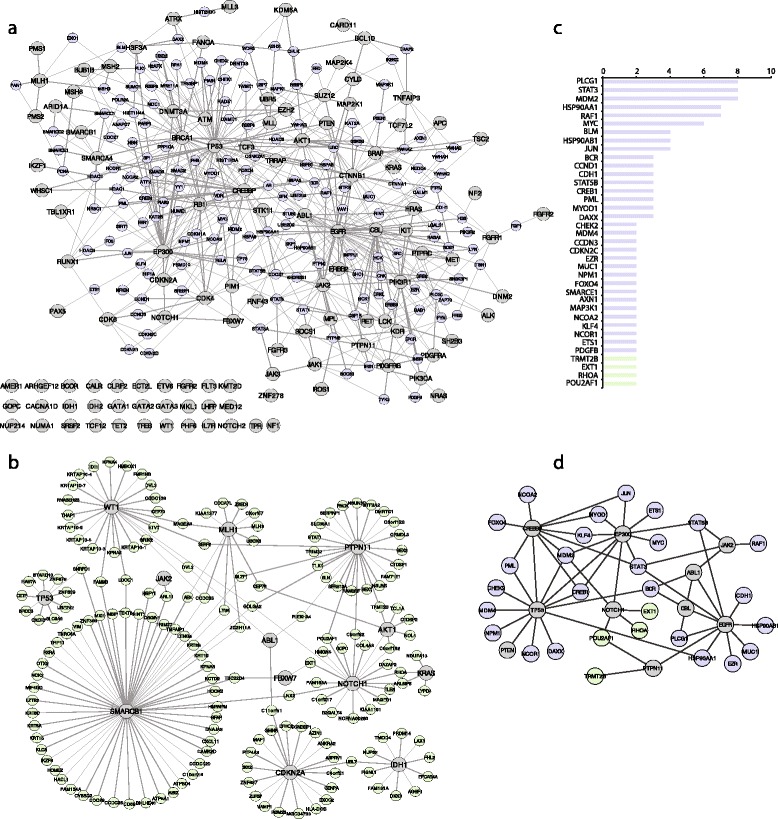
Interactome map of proteins involved in ALL and their partners (**a**) Literature curation of interactions between the 116 proteins mutated in ALL (*grey nodes*) and their human partners (*purple nodes*). **b** Interactions identified by high throughput Y2H screen. Cancer census gene encoded proteins are represented in *grey* and their partners in *green*. **c** The graph represents a ranking 37 candidates among the ALL-related partners based on the number of interactions between human proteins and ALL related proteins. In addition these genes are among the cancer gene census and they are expressed in 60 % of ALL-cell lines (*purple bars* represent literature-curated interactors and green bars represent Y2H interactors). The X-axis represents gene symbols; the Y-axis represents the number of partners. **d** Interactions between the 37 identified ALL-candidate genes and their partners among the ALL gene census

We hypothesized that the ALL-genes set is not limited to mutated genes in ALL samples, but could be extended to functional related genes and their products. In order to identify ALL-gene products interactors, we filtered from 3 different PPI databases (BioGRID, HPRD and IntAct), proteins that interact with at least 2 of the 116 ALL-gene products (Additional file 1: Table S5A). The obtained interactome map (Fig. 3a) shows that inter-connected ALL-gene products have also several common partners, prioritized according to the number of interacting ALL-gene products (Additional file 1: Table S5B). PPI stored in databases are curated from the literature and some proteins such as P53, BRCA1 or ATM heavily studied with hundreds of publications, have more PPI reported than others that are not studied with equal intensity. Previous studies suggested that unbiased PPI mapping allow characterization of overlooked PPI and identification of unknown diseases-related candidates [10]. Our previous interactome analysis for the ALL-gene products derive from database interrogations, we then performed an experimental yeast-hybrid (Y2H) unbiased PPI detection assay using a set of ALL-genes and the human ORFeome collection. We identified 193 interactions between 13 ALL gene products and 168 human partners. This experiment confirmed our observations using literature curated interactions, that interconnected ALL-gene products are also connected through several common partners in complex macromolecules (Fig. 3b). We identified several novel central hubs such as GOLGA2 that interacts with ALL-gene products NOTCH1, SMARCB1, PTPN11 and WT1, and CDC33, which is a common interactor of ALL-gene products MLH1, QT1, and SMARCB1 (Fig. 3b, Additional file 1: Table S4B).

As suggested by other studies, interconnected proteins are more associated with common diseases than expected by chance [40] and the same cancer driver genes are often involved in different cancer types, as evidenced by several examples [2]. To identify novel ALL-gene candidates through a “guilt-by-association” prediction, we prioritized ALL-gene products interactors using three criteria: (1) the number of ALL-gene products partners, (2) their implications in other types of cancer and (3) their expression in 24 common ALL cell lines. In total, we identified 37 ALL-gene products interactors that could be considered as ALL-associated candidates (Fig. 3c and d).

### EXT1 is functionally associated with the notch pathway through its interaction with NOTCH1 and FBXW7

**Fig. 4:**
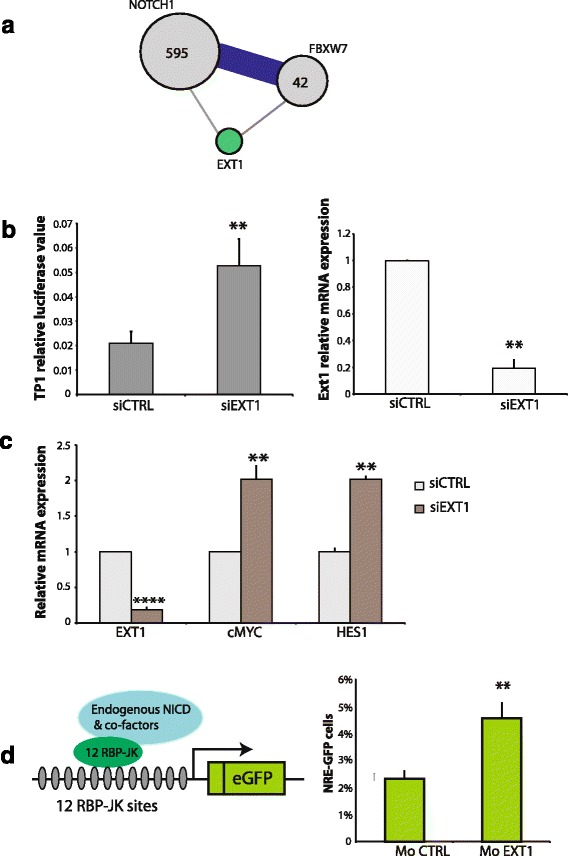
EXT1 depletion promotes NOTCH1 transcriptional activity. **a** Interactions between NOTCH1, EXT1 and FBXW7. Grey nodes represent proteins associated with ALL, with an area proportional to the number of distinct mutations. Bleu edges join pairs of interacting proteins for which mutations co-occur in the same samples. Edge widths are proportional to the number of samples with co-occurring mutations. The green node represent EXT1 and grey edges interactions identified in Y2H (**b**) Luciferase reporter assay using TP1-luciferase construct in HeLa Notch∆E-eGFP cell lines transfected with EXT1 siRNA or control siRNA as indicated. The relative luciferase values are normalized using a *Renilla* luciferase construct. Knock-down of EXT1 was analysed by qPCR. **c** mRNA expression levels of Notch1 target genes; cMYC and EXT1 analysed by qPCR following EXT1 Knock down. **d** A zebrafish transgenic line Tg (Tp1bglob:eGFP) um13, reporter for Notch1 transcriptional activity, were treated with control or *Ext1 a* and *c* ortholog-targeted morpholinos. Left panel represents *TP1 bglob:hngb1-eGFP* construct. The graph represents the percentage eGFP cells sorted by FACS. Data represent the means ± SEs of three independent experiments

The following example illustrates the validity of combining interactome approaches and gene mutations characterization to identify specific cancer type-related genes. Exostosin glycosyltransferase 1 (EXT1) is an endoplasmic reticulum transmembrane protein frequently mutated in multiple osteochondromas [41–43]. We identified EXT1 as a common interactor of two ALL-gene products NOTCH1 and FBW7 (Figs. 3b and 4a). We then investigated the potential functional interplay between EXT1 and the NOTCH pathway. Using NOTCH1 transcriptional-responsive luciferase reporter assay, we showed that depletion of *EXT1* using small interfering RNA increased NOTCH transactivation activity in different cell lines (Fig. 4b and Additional file 3: Figure S3a). We also showed that depletion of *EXT1* increases mRNA expression levels of two important NOTCH1-target genes: *HES1* and *MYC* (Fig. 4c). Consistent with this finding, over-expression of EXT1 inhibits NOTCH1-transactivation in different cell lines (Additional file 4: Figure S2) and correlates with a reduction of NICD protein levels (Additional file 5: Figure S4). We confirmed the effect of EXT1 on NOTCH1 pathway using a zebrafish in vivo model. We treated transgenic zebrafish line Tg (Tp1bglob:eGFP) um13 expressing fluorescent marker eGFP under the control of a Notch-responsive element TP1, with morpholinos targeting *EXT1*a and b zebrafish orhologs. As shown on Fig. 4d, we observed a 40 % increase of NOTCH1 activity following depletion of *EXT1* zebrafish orthologs (Fig. 4d).

**Fig. 5:**
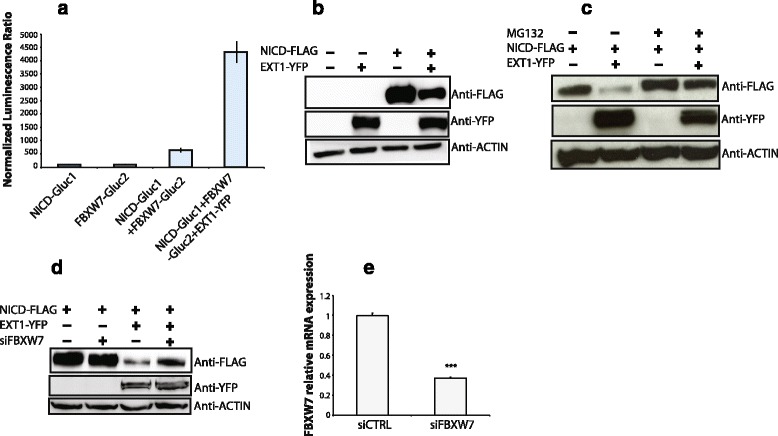
EXT1 regulates NOTCH1 degradation through FBXW7. **a** Protein complementation assay in HEK cells transfected with NICD-Gluc1 and/or FBXW7-Gluc2, in addition to EXT1-yfp as indicated in the X-axis, Normalized Luciferase Value (NLR) is represented by the Y-axis. **b**, (**c**) and (**d**) HEK293 cells were transfected with NICD-Flag and EXT1-YFP expressing plasmids as indicated. Twenty-four hours post-transfection cells were treated with proteasomal inhibitor MG132 for 6 h and lysates analyzed by western blot using anti-Flag M2 and anti-GFP antibodies. In (**d**) 24 h before overexpression, cells were transfected with siRNA for FBXW7 or a siRNA control using calcium phosphate. **e** Relative mRNA expression levels of *FBXW7* analyzed by qPCR. Data represent the means ± SEs of three independent experiments, each performed in triplicate

**Fig. 6:**
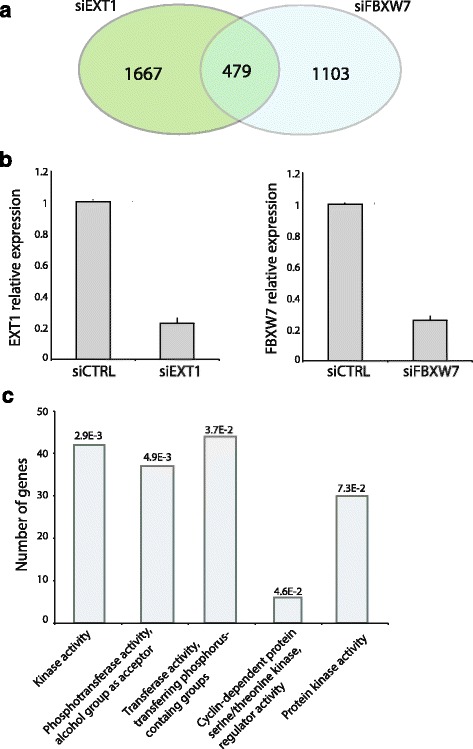
Genes coregulated by EXT1 and FBXW7. **a** HeLa Notch∆E-eGFP were treated siRNA for EXT1, FBXW7 or a control siRNA. Relative mRNA expression levels of *EXT1* and *FBXW7* were then analyzed by qPCR and RNA samples subjected to high throughput Illumina sequencing (RNA-seq). Ven diagrams represent a comparison between deregulated genes following knock down of EXT1 or FBXW7. **b** Relative mRNA expression levels of *EXT1* and *FBXW7* analyzed by qPCR. **c** Gene ontology enrichment analysis of common deregulated genes. GO-terms and node sizes are proportional to the number of genes implicated in the same GO term

FBXW7 is an E3 ubiquitin ligase regulating NOTCH1 proteasomal degradation [44]. To determine whether EXT1 interferes with FBXW7-NOTCH1 association, we first showed that the interaction between NOTCH1 and FBXW7 was dramatically enhanced in the presence of EXT1 (Fig. 5a). Then, we showed that, in the presence of EXT1, the level of NOTCH1 intracellular domain (NICD) is reduced in a proteasome-dependent manner (Fig. 5b and c: compare presence and absence of MG132 proteasome inhibitor). Interestingly, we also showed that reduced levels of NICD in the presence of EXT1 are FBXW7-dependent (Fig. 5d). To more deeply analyze the functional relationship between EXT1 and FBXW7 in regulating cellular homeostasis, a function well known for FBXW7 [44], we performed a genome-wide analysis of the transcriptome of HeLa-NICDdeltae-GFP [45] depleted for *EXT1* or *FBXW7*. We identified 479 mRNAs co-regulated by both EXT1 and FBXW7, which represent more than 30 % of FBXW7 targets (Fig. 6a). GO analysis of these EXT1/FBXW7 co-regulated genes finally indicated a significant enrichment in genes encoding for kinases including cyclin-dependent and MAP kinases (Fig. 6c and Additional file 1: Tables S6). Together, these results suggest that EXT1 is functionally linked to FBXW7, probably through priming kinases and substrates such as Notch1 towards proteasomal degradation.

## Conclusion

The sequencing of different cancer genomes allows identification and characterization of mutated genes in cancer samples. However, the development of genome-based therapies requires greater knowledge of the specific driver genes implicated in diverse cancer types and subtypes. In this study, we have analyzed mutated genes found in ALL samples collected in the Sanger COSMIC database. We proposed a list of genes more likely involved in ALL by combining the frequency of mutations in ALL samples, the number and pattern of distinct mutations, and the interconnectivity between their products that determine specific affected signaling pathways. We finally propose that novel ALL-gene candidates can be identified based on their functional association with well-known cancer genes. Finally, we demonstrated that EXT1, a tumor suppressor not previously linked to ALL, is involved in the regulation of the NOTCH pathway trough its dual interaction with NOTCH1 and FBXW7.

## Additional files

Additional file 1: Supplementary Tables. (XLSX 1285 kb) Additional file 2:
**Figure S1.** Distribution of mutations in ALL patients for NOTCH1, FLT3 and FBXW7. Schematic representation for each protein and its domains, the number of ALL samples with mutations localizations and its percentage for each protein are represented above each domain. Notch1 domains: Epidermal growth factor repeats (EGF repeats), Lin12 and Notch repeats (LNR), heterodimerization domain (HD), transmembrane domain (TD), RBP-Jk-associated module (RAM), ankyrin repeats (ANK), transactivation domain (TAD), proline, glutamic acid, serine, threonine-rich domain (PEST). FLT3 domains: extracellular domain (EC), transmembrane domain (TM), juxtamembrane (JM), amino-terminal and carboxy-terminal kinase domains (TK1 and TK2 respectively), kinase domain (K1), (STK). FBXW7 domains: tryptophan-aspartic acid 40 repeat (WD). (EPS 781 kb) Additional file 3: Figure S3. (a) EXT1 depletion promotes NOTCH1 transcriptional activity. (a) Luciferase reporter assay using TP1-luciferase construct in HEK293 cell lines transfected with EXT1 siRNA or control siRNA as indicated. The relative luciferase values are normalized using a *Renilla* luciferase construct. Knock-down of EXT1 was analysed by qPCR. (b) HEK293 cells were transfected with siRNA for *EXT1* or a siRNA control using calcium phosphate. Twenty-four hours post-transfection cells were transfected with NICD-Flag expressing plasmid as indicated, and after 24 h lysates analyzed by western blot using anti-Flag M2 antibody. Knock-down of EXT1 was analyzed by qPCR. (c) HeLaNotch1∆E-eGFP cells were transfected with EXT1 siRNA or control siRNA as indicated. Forty two hours pot-transfection, cells were treated with proteasomal inhibitor MG132 for 6 h and lysates were analyzed by western blot using a NICD antibody. Relative mRNA expression levels of *EXT1* analyzed by qPCR. (d) HeLaNotch1∆E-eGFP cells were transfected with EXT1-YFP expressing plasmid as indicated. Eighteen hours post transfection, cells were treated with proteasomal inhibitor MG132 for 6 h and lysates were analyzed by western blot using a NICD antibody. (EPS 3297 kb) Additional file 4: Figure S2. EXT1 inhibits Notch-1 transcriptional activation. (a) Luciferase reporter assay using TP1-luciferase construct in HEK293 and HeLa Notch∆E-eGFP cell lines transfected with PSG5C control plasmid, NICD or EXT1 constructs as indicated. The relative luciferase values are normalized using a *Renilla* luciferase construct. (b) Luciferase reporter assay using Gal4-luciferase construct in U2OS N1-Gal4 cell line transfected with PSG5C control plasmid or EXT1 construct and co-cultured with K562 control cells or K562 cells expressing Notch ligand DLL4 as indicated. Data represent the means ± SEs of three independent experiments, each performed in triplicate (EPS 752 kb) Additional file 5:
**Figure S4.** EXT1 reduces NOTCH1 levels in HelaNotch1∆EeGFP cells. Confocal images of HeLaNotch1∆E-eGFP cells transfected with EXT1-mCherry during time—lapse experiment. Green and red labeling corresponds to NOTCH1-GFP and EXT1-mCherry proteins localizations, respectively. Arrows indicate cells in which overexpression of EXT1 induced a decrease in GFP fluorescence. (PDF 6860 kb)
