# Supplementary figures and images for: Systematic interactome mapping of acute lymphoblastic leukemia cancer gene products reveals EXT-1 tumor suppressor as a Notch1 and FBWX7 common interactor
Source: BMC Cancer. 2016 May 26;16:335. doi: 10.1186/s12885-016-2374-2 (PMC4882867; doi:10.1186/s12885-016-2374-2)

**Figure S4**

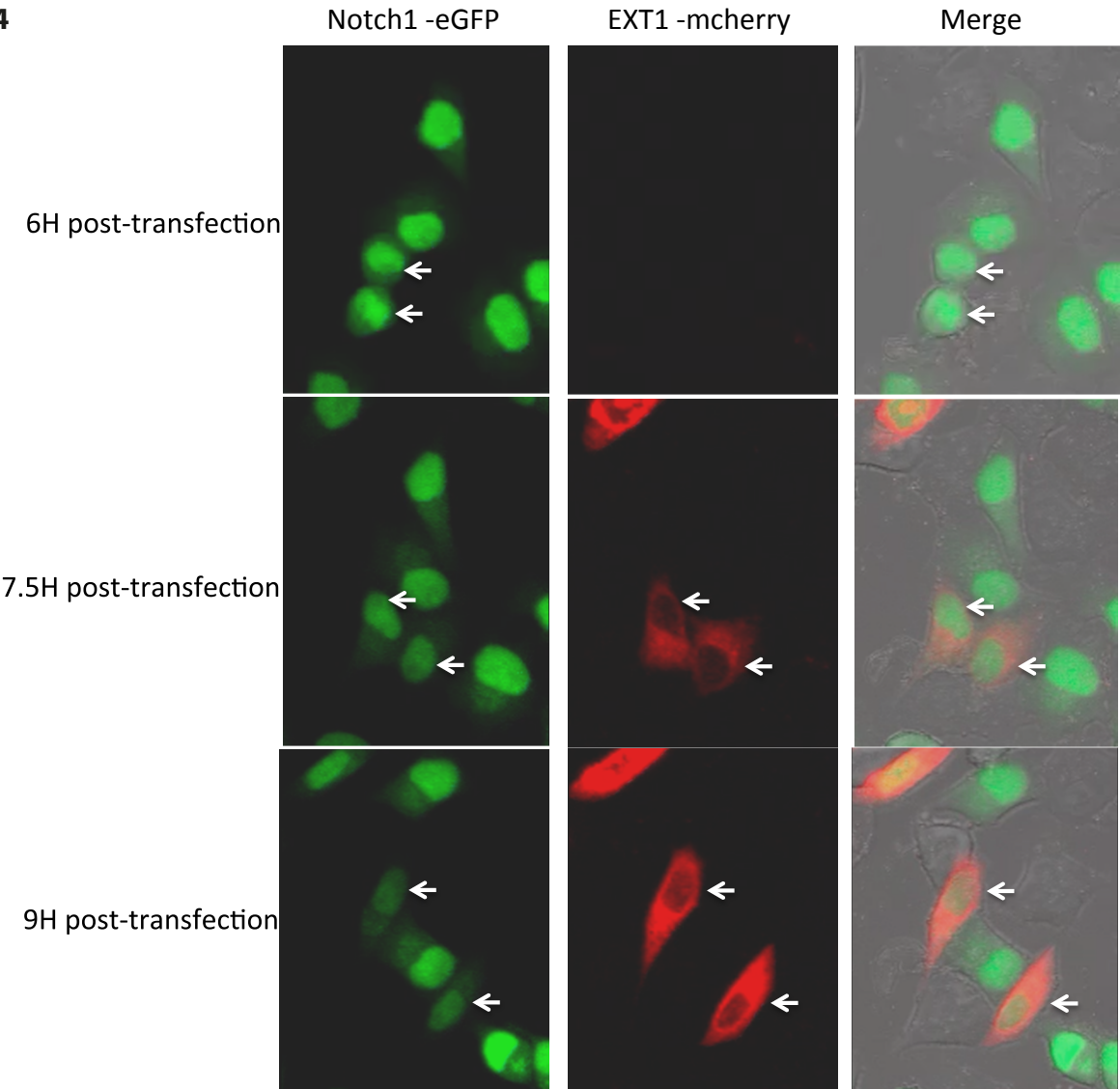

Supplement: Additional file 5: — Figure S4. EXT1 reduces NOTCH1 levels in HelaNotch1∆EeGFP cells. Confocal images of HeLaNotch1∆E-eGFP cells transfected with EXT1-mCherry during time—lapse experiment. Green and red labeling corresponds to NOTCH1-GFP and EXT1-mCherry proteins localizations, respectively. Arrows indicate cells in which overexpression of EXT1 induced a decrease in GFP fluorescence. (PDF 6860 kb) [file 12885_2016_2374_MOESM5_ESM.pdf]
